# More than meets the eye: a case of tubulointerstitial nephritis and uveitis syndrome

**DOI:** 10.1590/2175-8239-JBN-2026-0048en

**Published:** 2026-09-21

**Authors:** Telma Pais, Nadiesda Peres, Miguel Santos, Inês Leal, Joana Tavares, Sofia Jorge, José António Lopes, José Agapito Fonseca

**Affiliations:** 1 Unidade Local de Saúde Santa Maria, Lisboa, Portugal.

**Keywords:** Acute Tubulointerstitial Nephritis, Tubulointerstitial Nephritis and Uveitis, Acute Kidney Injury, Uveitis, Red Eye

## Abstract

Tubulointerstitial nephritis with uveitis (TINU) syndrome is a rare oculorenal inflammatory disorder characterized by concurrent tubulointerstitial nephritis (TIN) and uveitis in the absence of an alternative systemic disease. We present a 57-year-old woman with acute kidney injury, euglycemic glycosuria, and simul­taneous non-granulomatous anterior uveitis. Kidney biopsy demonstrated eosinophil-rich chronic active tubuloin­terstitial nephritis with non-caseating granulomas. After excluding infectious, drug-induced, and systemic autoimmune etiologies, TINU syndrome was diagnosed. Corticosteroid therapy resulted in substantial renal function recovery. This case underscores the diagnostic reasoning required to identify TINU syndrome in adult patients and illustrates the structured clinicopathological approach that enables timely, evidence-based management.

## Introduction

Tubulointerstitial nephritis and uveitis (TINU) syndrome is an immune-mediated oculorenal disorder defined by the coexistence of biopsy-confirmed tubulointerstitial nephritis (TIN) and uveitis in the absence of an alternative systemic disease accounting for both manifestations^1^. Approximately 600 cases have been described in the literature, yet the true incidence is likely underestimated due to incomplete ascertainment of asymptomatic or subclinical presentations^2,3 4^.

The syndrome demonstrates a predilection for children and adolescents, with a median age of onset of 17 years^2^. Adult-onset TIN, particularly from the fifth decade of life onward, is uncommon and therefore poses a diagnostic challenge. Its pathophysiology is incompletely understood but is presumed to involve an antigen-driven immune response, potentially triggered by infections or medications, superimposed on a background of genetic susceptibility mediated by specific HLA alleles^4,5,6^.

Beyond its rarity, this case offers an opportunity to revisit the diagnostic approach to an uncommon but treatable condition: recognizing the oculorenal association, systematically excluding alternative diagnoses, correlating histopathological findings with the clinical picture, and tailoring therapy to dual-organ involvement.

## Diagnostic Reasoning

### Clinical Presentation and Initial Assessment

A 57-year-old woman with no prior medical history was referred to Nephrology for fatigue and acute kidney injury (AKI). Serum creatinine was 3.54 mg/dL and serum urea was 130 mg/dL, compared with a previously normal creatinine level of 0.9 mg/dL documented four years prior. The absence of a personal or family history of renal disease, systemic symptoms, and the lack of any regular medication use or recent drug or supplement exposure was a notable negative finding.

The urinary profile revealed subnephrotic proteinuria (urine protein-to-creatinine ratio of 774 mg/g) with a disproportionately low urine albumin-to-creatinine ratio of 162 mg/g, consistent with tubular rather than glomerular proteinuria. Euglycemic glycosuria was present, a hallmark of proximal tubular dysfunction, along with bland urinary sediment. Renal ultrasound showed diffuse parenchymal hyperechogenicity with no evidence of urinary obstruction.

**Figure 1:**
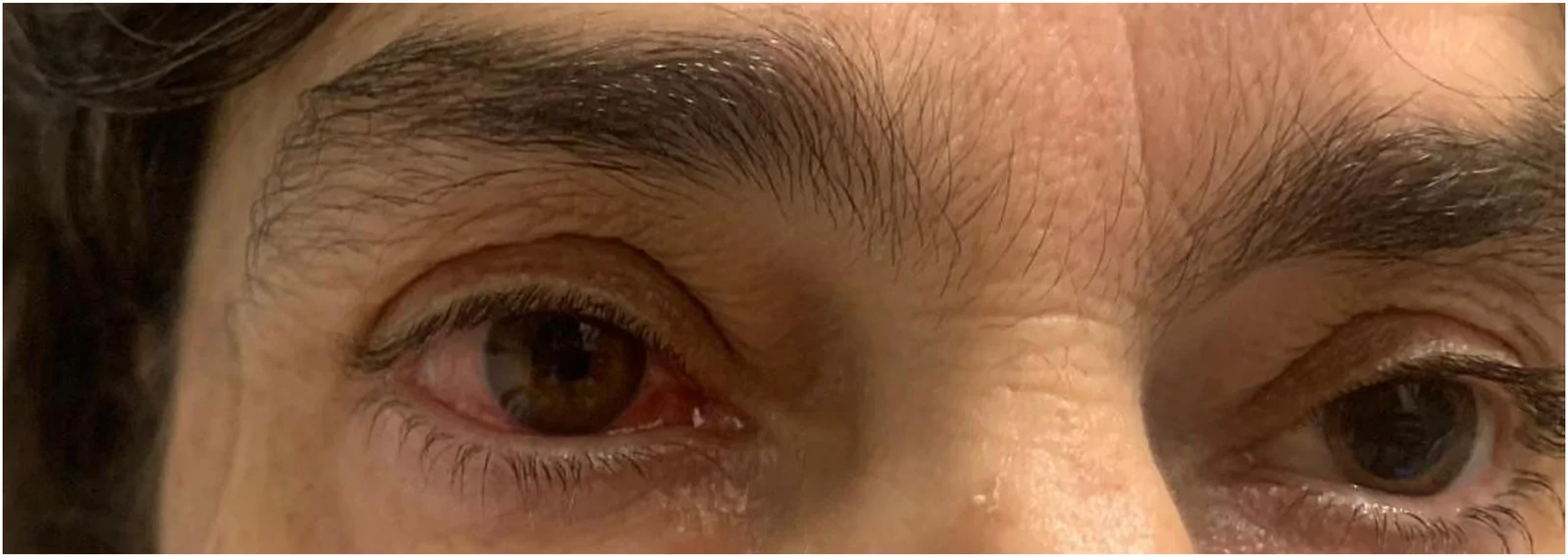
Red eye at presentation to the Nephrology visit.

At the initial Nephrology visit, the patient presented with a one-week history of right ocular pain and redness (Figure 1). Physical examination was otherwise unremarkable. Urgent ophthalmological assessment confirmed acute non-granulomatous anterior uveitis in the right eye, and she was started on topical ocular cyclopentolate and prednisolone. The temporal co-occurrence of AKI with proximal tubular dysfunction and acute anterior uveitis, without systemic features pointing toward an alternative diagnosis, raised clinical suspicion for TINU syndrome.

### Laboratory Evaluation and Differential Diagnosis

Laboratory workup (Table 1) demonstrated normocytic normochromic anemia, mild eosinophilia, and an elevated erythrocyte sedimentation rate (70 mm/h), consistent with a systemic inflammatory state. Rheumatoid factor was mildly elevated; however, anti-nuclear antibodies (ANA), anti-dsDNA, ANCA (PR3 and MPO), and complement levels were within normal limits. Serum and urine immunofixation results were negative.

**Table 1:** Laboratory results at presentation

Tests	Results	Reference range
Hemoglobin, g/dL	11.3	13–17.5
Leukocytes, x109/L	9.7	4.0–11.0
Eosinophils, x109/L	0.55	0.0–0.5
Platelets, x109/L	280	150–450
Serum urea, mg/dL	130	16–49
Serum creatinine, mg/dL	3.54	0.5–0.9
Serum albumin, g/dL	4.4	3.5–5.2
Erythrocyte sedimentation rate, mm/h	70	<20
C-reactive protein, mg/dL	0.83	<0.5
Rheumatoid factor, IU/mL	47.2	<20
Anti-neutrophil cytoplasmic antibodies PR3, UQ	Negative	–
Anti-neutrophil cytoplasmic antibodies MPO, UQ	Negative	–
Anti-nuclear antibodies	Negative	–
Anti-double-stranded DNA antibodies, IU/mL	Negative	–
C3 complement, mg/dL	136	90–180
C4 complement, mg/dL	55	10–40
Angiotensin-converting enzyme, U/Q	43	8–52
Serum protein electrophoresis	Normal	–
Serum and urine immunofixation	Negative	–

The differential diagnosis was systematically addressed. Systemic autoimmune diseases—including systemic lupus erythematosus (negative ANA and anti-dsDNA), Sjögren’s disease, sarcoidosis (angiotensin-converting enzyme [ACE] within the normal range), and granulomatosis with polyangiitis (negative ANCA)—were rendered unlikely by the serological and clinical profile. Infectious etiologies, including tuberculosis, toxoplasmosis, and brucellosis, were considered but were not supported by the clinical history or laboratory data. Drug-induced TIN was excluded by the confirmed absence of any nephrotoxic exposure. Given the coexistence of tubular proteinuria and glycosuria, Fanconi syndrome secondary to a plasma cell dyscrasia was also considered and excluded by normal results on serum protein electrophoresis and negative immunofixation.

Given that TINU syndrome is fundamentally a diagnosis of exclusion, the systematic elimination of these alternative etiologies was necessary before it could be established with confidence.

## Clinical Challenges

### Adult-Onset Presentation

The most immediate diagnostic challenge in this case was the patient’s age. TINU syndrome predominantly affects children and adolescents^2^. Adult-onset cases have also been reported, although they are less common and show a slight predominance in women^7,8,9^. The present case reinforces that age should not serve as an exclusionary criterion.

### Asynchronous Oculorenal Manifestations

A well-recognized feature of TINU syndrome is the temporal dissociation between renal and ocular involvement^3,8^. Uveitis typically occurs between two months before and up to twelve months after the onset of kidney disease, though the two manifestations may overlap^8^. In the present case, ocular and renal symptoms were concurrent, which, while diagnostically convenient, is not the most common presentation. When uveitis presents in isolation, the diagnosis of TINU may not be initially considered because nephritis may be absent; when only AKI is present, the ocular component may be overlooked or remain subclinical^10^. This underscores the importance of proactive ophthalmological evaluation in all patients with unexplained AKI and inflammatory features, even in the absence of overt ocular complaints.

### Diagnostic Uncertainty and The Role of Kidney Biopsy

In the absence of a pathognomonic biomarker, kidney biopsy remains the cornerstone of diagnosis. In this patient, renal biopsy was pursued, given the severity of AKI and the absence of a clear clinical diagnosis, which supported the need to characterize the renal lesion.

## Clinicopathological Correlation

### Renal Histopathology

**Figure 2:**
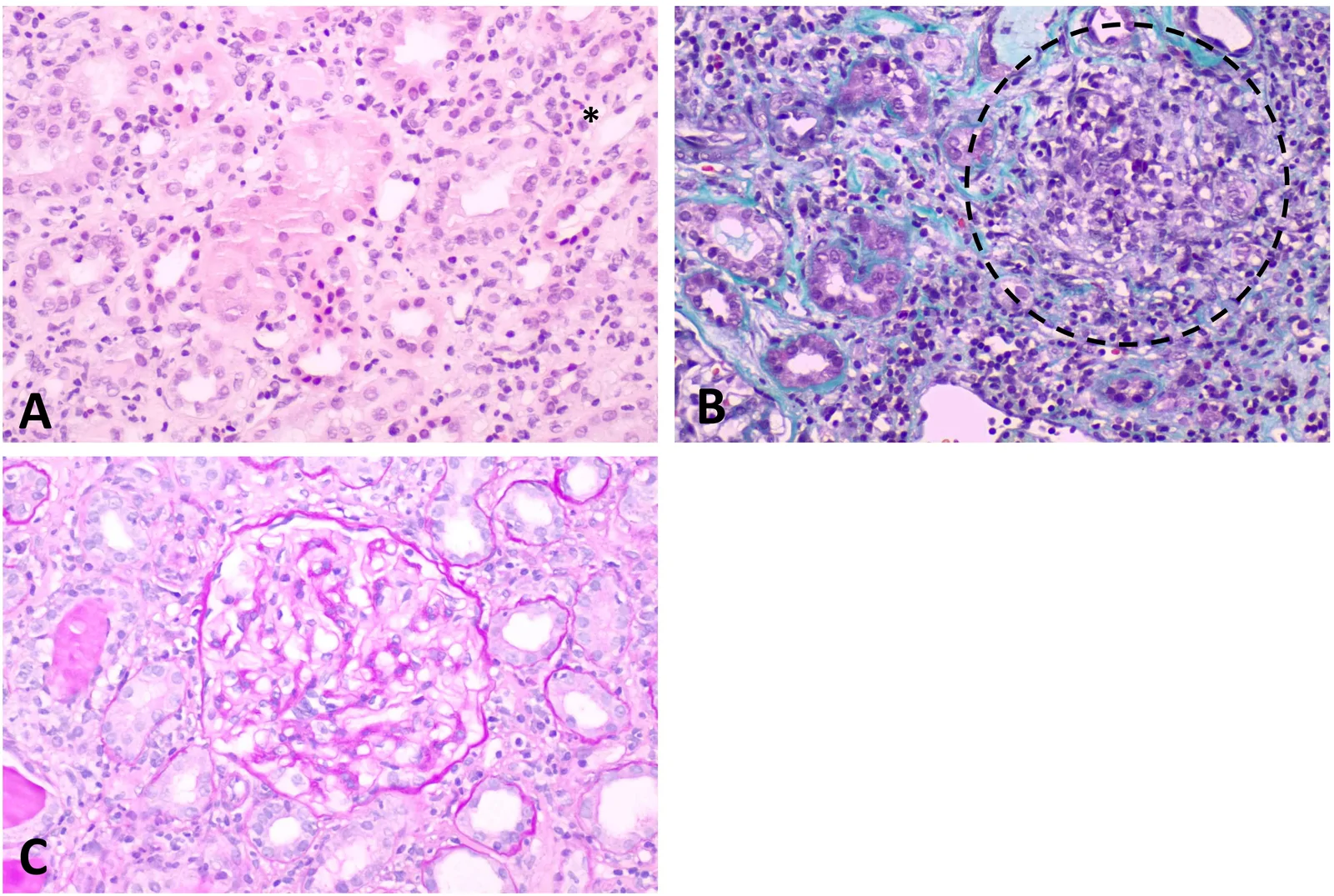
Kidney biopsy. A. [Hematoxylin & Eosin, 200×] Degenerative changes in the cortical tubular epithelium are associated with a mild to moderate chronic active inflammatory infiltrate; note the presence of eosinophils (*). B. [Masson’s trichrome, 200×] Focally, there are aggregates of histiocytic cells, with one area showing a structure suggestive of a non-necrotizing granuloma (dashed circle). C. [Periodic acid-Schiff (PAS), 200×] The glomerular structure is preserved.

The kidney biopsy demonstrated moderately active chronic tubulointerstitial nephritis (Figure 2, A–2) characterized by three key findings: (i) a predominantly eosinophil-rich inflammatory infiltrate; (ii) non-caseating granuloma formation, consisting of aggregates of histiocytic cells; and (iii) mild interstitial fibrosis with extensive degenerative tubular changes and intraluminal granular debris.

Characteristic kidney biopsy findings in TINU syndrome include tubulointerstitial edema and an inflammatory cell infiltrate, mainly composed of mononuclear cells^8^. Eosinophils may be present, although they are more commonly associated with drug-induced TIN, highlighting the importance of excluding this etiology^11^. Non-caseating granulomas within the tubulointerstitium raise the differential diagnosis of sarcoidosis; however, normal serum ACE levels, the absence of systemic granulomatous disease, and the non-granulomatous nature of the concurrent uveitis (non-granulomatous anterior uveitis is the predominant ocular phenotype in TINU, whereas sarcoid uveitis characteristically presents as granulomatous anterior uveitis) argued against this diagnosis^11,12^.

Glomerular architecture was preserved on PAS staining, and immunofluorescence (not shown) did not reveal immune complex deposits, findings that are consistent with TINU. The presence of mild interstitial fibrosis, coexisting with active inflammation, suggested a subacute process.

### Tubular Dysfunction and Proximal Tubular Injury

Renal involvement in TINU syndrome typically manifests as AKI; proteinuria, sterile pyuria, and microscopic hematuria are commonly observed. When proximal tubular injury is prominent, as in this patient, additional features of tubular dysfunction may emerge. Euglycemic glycosuria reflects global proximal tubular epithelial injury, impairing the reabsorptive capacity of the proximal tubule. The apparent disproportionate contribution of tubular (non-albumin) protein to the total urinary protein- to-creatinine ratio further supports significant proximal tubular dysfunction. These features suggest a partial Fanconi syndrome, a recognized manifestation of TIN, consistent with the histological degenerative changes observed^13^.

### Ocular Pathology

The non-granulomatous anterior uveitis confirmed on ophthalmological evaluation is the predominant ocular manifestation of TINU syndrome, occurring in over 80% of affected patients^14^. While initially unilateral in many cases, bilateral involvement (either synchronous or metachronous) occurs in up to 88% of patients^2^.

## Diagnostic and Therapeutic Implications

### Establishing the Diagnosis

TINU syndrome requires histological confirmation of TIN and clinical confirmation of uveitis following the systematic exclusion of alternative etiologies. The diagnostic criteria proposed by Mandeville et al. stratify cases into definite, probable, and possible TINU based on the combination of biopsy findings and urinalysis abnormalities, acknowledging that biopsy is not always feasible^1^. In the present case, the diagnosis was considered definite, established by biopsy-confirmed TIN and ophthalmologically verified anterior uveitis in the absence of any concurrent systemic disease.

### Therapeutic Strategy

Treatment of TINU syndrome is directed at organ-specific involvement. Systemic corticosteroid therapy remains the mainstay of treatment for patients with significant renal involvement. In keeping with first-line recommendations, this patient received prednisolone at 1 mg/kg/day, tapered rapidly to 20 mg by week three, and then gradually withdrawn over a total of 16 weeks. This duration was consistent with the typically recommended three-to-six-month course, depending on treatment response^3^. This prolonged regimen contrasts with the shorter courses employed in drug-induced acute interstitial nephritis, where corticosteroids are generally tapered over four to six weeks.

The rationale for corticosteroid therapy in TINU is supported by emerging evidence challenging the historical assumption that kidney disease in this syndrome is self-limited. While early case series suggested that the recovery of kidney function was independent of systemic corticosteroid use, more recent data, including serial biopsy studies, indicate that incomplete renal recovery and progression to chronic kidney disease may occur^15,16^. This case reinforces the importance of prompt pharmacological intervention in adults with significant AKI.

Uveitis tends to respond to corticosteroids, but recurrence is common and may occur independently of renal disease activity. In this patient, following withdrawal of systemic corticosteroids, asymptomatic ocular inflammation developed in the contralateral eye. Optical coherence tomography and fluorescein angiography were unremarkable. Topical prednisolone was initiated with a good clinical response.

Topical corticosteroids and cycloplegic agents are first-line treatments for anterior uveitis; severe or non-anterior disease warrants systemic corticosteroids or steroid-sparing immunomodulatory therapy. The same second-line agents, such as methotrexate, azathioprine, or mycophenolate mofetil, are also used when renal disease fails to respond adequately to corticosteroids alone. TNF-alpha inhibitors have demonstrated efficacy in severe or treatment-refractory cases^17^.

### Prognosis and Follow-up

**Figure 3:**
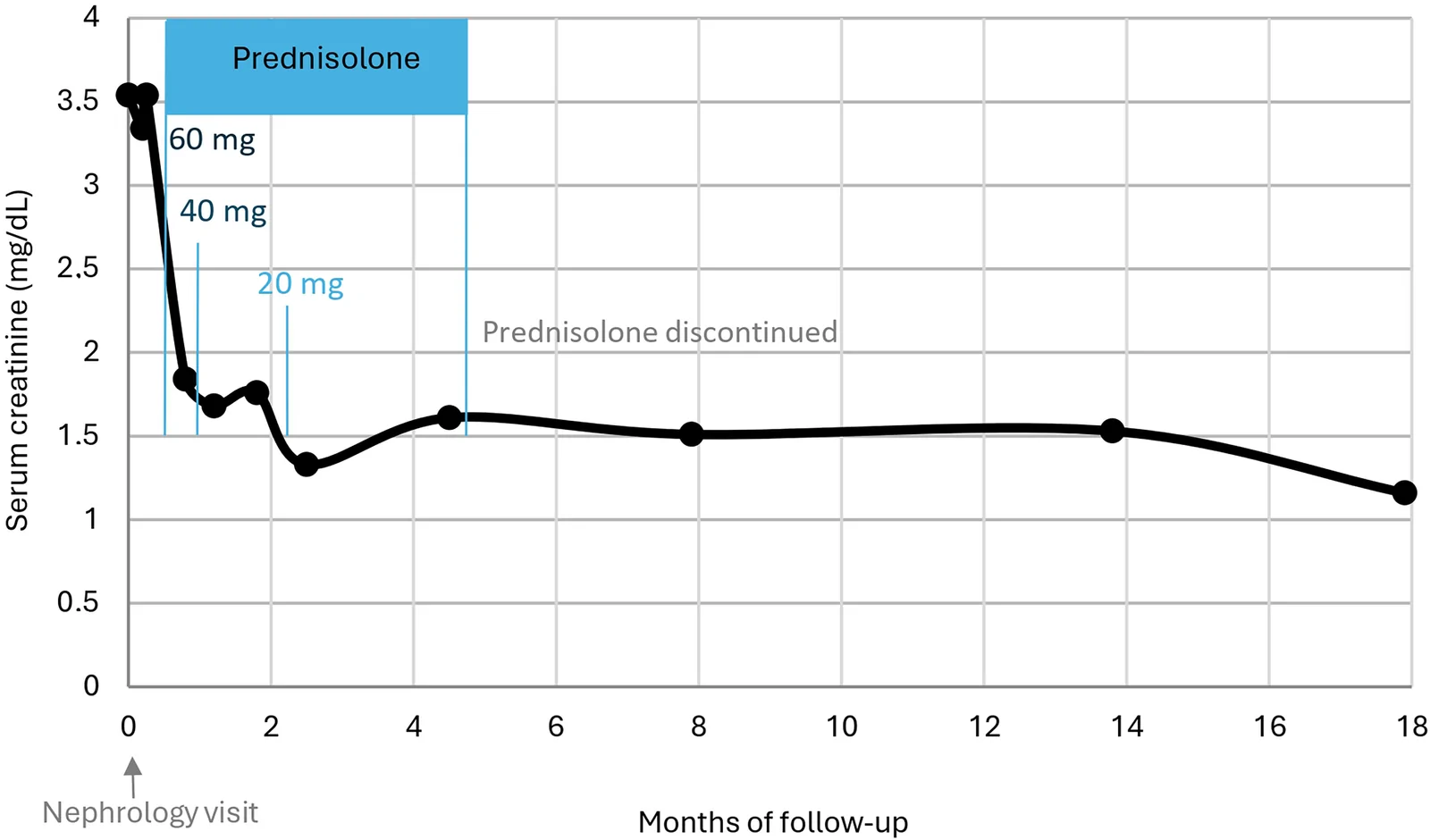
Trends in serum creatinine levels (solid line) and corticosteroid treatment during follow-up.

In this patient, serum creatinine improved from 3.54 mg/dL at presentation to 1.61 mg/dL after completing steroid therapy, and further to 1.15 mg/dL at the 10-month follow-up (Figure 3), with normalization of the urinary albumin-to-creatinine ratio (30 mg/g) and resolution of glycosuria. This favorable trajectory is consistent with the relatively mild interstitial fibrosis on biopsy.

Prognosis is usually favorable when treatment is initiated promptly. Prognostic risk stratification in TINU is informed by several factors: adult age at diagnosis, presence of posterior uveitis or panuveitis, and degree of chronicity on renal biopsy are associated with an increased risk of CKD^2^. Children tend to have more frequent uveitis relapses, but a lower risk of kidney injury^2^. Permanent renal dysfunction occurs in up to 15% of patients, while chronic ocular complications (most frequently posterior synechiae) affect approximately 20%^8,18^. Long-term multidisciplinary surveillance by both Nephrology and Ophthalmology is therefore essential.

## Conclusion

In conclusion, this case highlights the importance of maintaining a high index of suspicion for TINU syndrome whenever altered kidney function and ocular inflammation coexist, whether presenting concomitantly or sequentially. The oculorenal association is the key diagnostic clue, and proactive ophthalmological evaluation should be considered in any patient with unexplained AKI and features of proximal tubular dysfunction, irrespective of age.

Multidisciplinary collaboration between Nephrology and Ophthalmology remains the cornerstone of optimal patient care in TINU syndrome, and timely diagnosis is the critical first step toward preserving both renal function and visual outcomes.

## Data Availability

No datasets were generated or analyzed during the preparation of this case report. All relevant data are contained within the manuscript.
