# Supplementary material for: More than meets the eye: a case of tubulointerstitial nephritis and uveitis syndrome
Source: J Bras Nefrol. 2026 Sep 21;48(3):e20260048. doi: 10.1590/2175-8239-JBN-2026-0048en (PMC13599237; doi:10.1590/2175-8239-JBN-2026-0048en)
Supplement: Translation

# Translation: Mais do que aparenta: um caso de síndrome de nefrite tubulointersticial e uveíte

**DOI:** 10.1590/2175-8239-JBN-2026-0048pt

**Authors:** Telma Pais, Nadiesda Peres, Miguel Santos, Inês Leal, Joana Tavares, Sofia Jorge, José António Lopes, José Agapito Fonseca

**Affiliations:** 1 Unidade Local de Saúde Santa Maria, Lisboa, Portugal.

**Keywords:** Nefrite Tubulointersticial Aguda, Nefrite Tubulointersticial e Uveíte, Lesão Renal Aguda, Uveíte, Olho Vermelho

## Abstract

A síndrome de nefrite tubulointersticial e uveíte (TINU) é uma patologia inflamatória oculorrenal rara, caracte­rizada pela ocorrência concomitante de nefrite tubulointersticial (NTI) e uveíte, na ausência de outra doença sistêmica que justifique ambas as manifestações. Apresentamos o caso de uma mulher de 57 anos com lesão renal aguda, glicosúria normoglicêmica e uveíte anterior não granulomatosa concomitante. A biópsia renal revelou nefrite tubulointersticial crônica ativa, rica em eosinófilos, com granulomas não caseosos. Após exclusão de etiologias infecciosas, medicamentosas e autoimunes sistêmicas, foi estabelecido o diagnóstico de síndrome TINU. O tratamento com corticoterapia resultou em recuperação substancial da função renal. Este caso evidencia o raciocínio diagnóstico necessário para identificar a síndrome TINU em pacientes adultos e demonstra uma abordagem clínico-patológica estruturada que permitiu um manejo oportuno, baseado em evidências.

## Introdução

A síndrome de nefrite tubulointersticial e uveíte (TINU) é uma doença oculorrenal imunomediada, definida pela coexistência de nefrite tubulointersticial (NTI) con­firmada por biópsia renal e uveíte, na ausência de outra doença sistêmica que explique ambas as manifestações clínicas^1^. Encontram-se descritos na literatura aproximadamente 600 casos; no entanto, a verdadeira incidência é provavelmente subestimada devido às limitações no diagnóstico de apresentações assintomáticas ou subclínicas^2,3,4^.

A síndrome manifesta-se predomi­nantemente em crianças e adolescentes, com uma idade mediana de início de 17 anos^2^. O aparecimento de NTI em adultos, particularmente a partir da quinta década de vida, é incomum e constitui um desafio diagnóstico. A sua fisiopatologia não se encontra completamente esclarecida, mas acredita-se que envolva uma resposta imunitária contra antígenos, potencialmente desencadeada por infecções ou fármacos, em indivíduos com maior suscetibilidade genética associada a alguns alelos do complexo HLA^4,5,6^.

Este caso constitui uma oportunidade para rever a abordagem diagnóstica de uma entidade incomum, mas potencialmente tratável: reconhecer a associação oculorrenal, excluir sistematicamente diagnósticos alternativos, correlacionar os achados histopatológicos com o contexto clínico e adequar a terapia ao envolvimento simultâneo de ambos os órgãos.

## Raciocínio Diagnóstico

### Apresentação Clínica e Avaliação Inicial

Uma mulher de 57 anos, sem antecedentes médicos relevantes, foi encaminhada para a consulta de Nefrologia por fadiga e lesão renal aguda (LRA). Apresentava creatinina sérica de 3,54 mg/dL e ureia sérica de 130 mg/dL, em comparação com um valor prévio de creatinina de 0,9 mg/dL, documentado quatro anos antes. Destaca-se a ausência de antecedentes pessoais ou familiares de doença renal, de sintomas sistêmicos, de medicação habitual, ou de exposição recente a fármacos ou suplementos.

A avaliação urinária revelou proteinúria subnefrótica (relação proteinúria/creatinúria de 774 mg/g), com uma relação albuminúria/creatinúria desproporcionalmente baixa (162 mg/g), compatível com proteinúria de origem tubular e não glomerular. Observou-se ainda glicosúria normoglicêmica, um achado característico de disfunção tubular proximal, associada a um sedimento urinário inativo. A ultra­ssonografia renal demonstrou hiperecogenicidade difusa do parênquima, sem evidência de obstrução das vias urinárias.

Na consulta inicial de Nefrologia, a paciente apresentava história de uma semana de dor e hiperemia ocular à direita (Figura 1), sem outras alterações de relevo no exame físico. A avaliação urgente por Oftalmologia confirmou uma uveíte anterior aguda não granulomatosa do olho direito, tendo sido iniciada terapia tópica com ciclopentolato e prednisolona. A ocorrência simultânea de LRA, disfunção tubular proximal e uveíte anterior aguda, na ausência de manifestações sistêmicas sugestivas de um diagnóstico alternativo, levantou a suspeita clínica de síndrome TINU.

**Figura 1:** Hiperemia ocular do olho direito na primeira consulta de Nefrologia.

### Avaliação Laboratorial e Diagnóstico Diferencial

O estudo analítico (Tabela 1) revelou anemia normocítica normocrômica, eosinofilia leve e veloci­dade de hemossedimentação aumentada (70 mm/h), compatíveis com um estado inflamatório sistêmico. O fator reumatoide encontrava-se ligeiramente elevado; os anticorpos antinucleares (ANA), anti-dsDNA, ANCA (PR3 e MPO) e os níveis de complemento encontravam-se dentro dos valores de referência. A imunofixação sérica e urinária foi negativa.

**Tabela 1:** Resultados laboratoriais na apresentação

Testes	Resultados	Valores de referência
Hemoglobina, g/dL	11,3	13–17,5
Leucócitos, x10^9^/L	9,7	4,0–11,0
Eosinófilos, x10^9^/L	0,55	0,0–0,5
Plaquetas, x10^9^/L	280	150–450
Ureia sérica, mg/dL	130	16–49
Creatinina sérica, mg/dL	3,54	0,5–0,9
Albumina sérica, g/dL	4,4	3,5–5,2
Velocidade de hemossedimentação, mm/h	70	<20
Proteína C reativa, mg/dL	0,83	<0,5
Fator reumatoide, UI/mL	47,2	<20
Anticorpos citoplasmáticos anti-neutrófilos PR3, UQ	Negativa	–
Anticorpos citoplasmáticos anti-neutrófilos MPO, UQ	Negativa	–
Anticorpos antinucleares	Negativa	–
Anticorpos anti-DNA de dupla hélice, UI/mL	Negativa	–
Complemento C3, mg/dL	136	90–180
Complemento C4, mg/dL	55	10–40
Enzima conversora da angiotensina, U/Q	43	8–52
Eletroforese de proteínas séricas	Normal	–
Imunofixação sérica e urinária	Negativa	–

O diagnóstico diferencial foi abordado de forma sistemática. As doenças autoimunes sistêmicas — incluindo lúpus eritematoso sistêmico (ANA e anti-dsDNA negativos), doença de Sjögren, sarcoidose (enzima conversora da angiotensina [ECA] dentro dos valores de referência) e granulomatose com poliangeíte (ANCA negativos) — foram consideradas pouco prováveis com base no perfil clínico e sorológico. Etiologias infecciosas, incluindo tuberculose, toxoplasmose e brucelose, também foram avaliadas, mas se mostraram pouco prováveis com base na história clínica e nos resultados laboratoriais. A NTI induzida por fármacos foi excluída devido à ausência de exposição a agentes potencialmente nefrotóxicos. Atendendo à coexistência de proteinúria tubular e glicosúria, considerou-se ainda a hipótese de síndrome de Fanconi secundária a discrasia plasmocitária, posteriormente excluída pela eletroforese de proteínas séricas normal e pela imunofixação negativa.

Dado que a síndrome TINU é, por definição, um diagnóstico de exclusão, a eliminação sistemática destas etiologias alternativas foi essencial antes de o diagnóstico poder ser estabelecido com confiança.

## Desafios Clínicos

### Apresentação na Idade Adulta

O primeiro desafio diagnóstico neste caso esteve relacionado à idade da paciente. A síndrome TINU é predominantemente uma doença da infância e da adolescência^2^. Embora menos frequentes, casos de início na idade adulta têm sido descritos, com leve predomínio no sexo feminino^7,8,9^. O presente caso reforça que a idade não deve constituir um critério de exclusão do diagnóstico.

### Manifestações Oculorrenais Assíncronas

Uma característica bem reconhecida da síndrome TINU é a possível dissociação temporal entre o envolvimento renal e ocular^3,8^. A uveíte pode surgir desde dois meses antes até doze meses após o início da doença renal, embora ambas as manifestações possam ocorrer simultaneamente^8^. Neste caso, os sintomas oculares e renais foram concomitantes, o que facilitou o diagnóstico, embora esta não seja a apresentação mais frequente. Quando a uveíte ocorre isoladamente, o diagnóstico de síndrome TINU pode não ser inicialmente considerado, uma vez que a nefrite pode ainda não estar presente; por outro lado, quando há apenas LRA, o envolvimento ocular pode passar despercebido ou permanecer subclínico^10^. Esse fato sublinha a importância de uma avaliação oftalmológica sistemática em todos os pacientes com LRA de etiologia não esclarecida e evidência de inflamação, mesmo na ausência de queixas oculares.

### Incerteza Diagnóstica e Papel da Biópsia Renal

Dado que não existe um biomarcador patogno­mônico, a biópsia renal permanece o pilar do diagnóstico. Nesta paciente, a gravidade da LRA e a ausência de um diagnóstico clínico claro justificaram a realização de uma biópsia renal para caracterizar mais detalhadamente a lesão renal presente.

## Correlação Clínico-Patológica

### Histopatologia Renal

A biópsia renal demonstrou nefrite tubulointersticial crônica moderadamente ativa (Figura 2, A–C), caracterizada por três achados principais: (i) infiltrado inflamatório predominantemente rico em eosinófilos; (ii) formação de granulomas não caseosos, constituídos por agregados de histiócitos; e (iii) fibrose intersticial leve, associada a alterações degenerativas tubulares extensas e detritos granulares intraluminais.

**Figura 2:** Biópsia renal. **A.** [Hematoxilina & Eosina, 200×] As alterações degenerativas do epitélio tubular cortical associam-se a um infiltrado inflamatório crônico ativo leve a moderado; destaca-se a presença de eosinófilos (*). **B.** [Tricrômio de Masson, 200×] Observam-se, focalmente, agregados de histiócitos, com uma área que demonstra uma estrutura sugestiva de granuloma não necrosante (círculo tracejado). **C.** [Ácido periódico de Schiff (PAS), 200×] A estrutura glomerular encontra-se preservada.

Os achados histológicos característicos da síndrome TINU incluem edema tubulointersticial e infiltrado inflamatório, predominantemente constituído por células mononucleares^8^. Os eosinófilos podem estar presentes, embora estejam mais frequentemente associados à NTI induzida por fármacos, o que reforça a importância da exclusão dessa etiologia^11^. A presença de granulomas não caseosos leva ao diagnóstico diferencial com sarcoidose; contudo, os níveis séricos normais de ECA, a ausência de doença granulomatosa sistêmica e o caráter não granulomatoso da uveíte concomitante — sendo a uveíte anterior não granulomatosa o fenótipo ocular predominante na síndrome TINU, enquanto a uveíte da sarcoidose é tipicamente granulomatosa — tornaram esse diagnóstico pouco provável^11,12^.

A arquitetura glomerular encontrava-se preservada na coloração PAS, e o estudo por imunofluorescência (não apresentado) não identificou depósitos imunes, achados compatíveis com a síndrome TINU. A coexistência de fibrose intersticial leve com inflamação ativa era sugestiva de um processo subagudo.

### Disfunção Tubular e Lesão Tubular Proximal

O envolvimento renal na síndrome TINU manifesta-se habitualmente como LRA, sendo a presença de proteinúria, leucocitúria estéril e hematúria microscópica frequentemente descrita. Quando a lesão tubular proximal é significativa, como neste caso, podem surgir manifestações adicionais de disfunção tubular. A glicosúria normoglicêmica reflete lesão global do epitélio tubular proximal, comprometendo a capacidade de reabsorção desse segmento do néfron. O predomínio aparente de proteinúria de origem tubular apoia a existência de disfunção tubular proximal significativa. Esses achados sugerem uma síndrome de Fanconi parcial, uma manifestação reconhecida de NTI, consistente com as alterações degenerativas observadas na biópsia^13^.

### Patologia Ocular

A uveíte anterior não granulomatosa confirmada na avaliação oftalmológica constitui a manifestação ocular predominante da síndrome TINU, ocorrendo em mais de 80% dos pacientes afetados^14^. Embora inicialmente unilateral em muitos casos, o envolvimento bilateral (síncrono ou metacrônico) ocorre em até 88% dos pacientes^2^.

## Implicações Diagnósticas e Terapêuticas

### Estabelecer o Diagnóstico

A síndrome TINU requer confirmação histológica de NTI e confirmação clínica de uveíte, após exclusão sistemática de etiologias alternativas. Os critérios diagnósticos propostos por Mandeville et al. classificam os casos em TINU definitiva, provável e possível, com base na combinação dos achados histológicos e das alterações urinárias, reconhecendo que a realização de biópsia nem sempre é factível^1^. Neste caso, o diagnóstico foi considerado definitivo, estabelecido pela confirmação histológica de NTI e pela uveíte anterior confirmada pela Oftalmologia, na ausência de qualquer doença sistêmica concomitante.

### Estratégia Terapêutica

O tratamento da síndrome TINU é dirigido ao envolvimento específico de cada órgão. A corticoterapia sistêmica mantém-se como a terapia de primeira linha em pacientes com envolvimento renal significativo. De acordo com essas recomen­dações, a paciente iniciou prednisolona na dose de 1 mg/kg/dia, com redução rápida para 20 mg na terceira semana e diminuição progressiva ao longo de 16 semanas. Essa duração foi consistente com o curso terapêutico habitualmente recomendado de três a seis meses, ajustado à resposta clínica^3^. Este regime prolongado contrasta com os esquemas mais curtos utilizados na NTI induzida por fármacos, nos quais a corticoterapia é geralmente reduzida ao longo de quatro a seis semanas.

A utilização de corticoterapia na síndrome TINU baseia-se em dados que demonstraram que a doença renal não parece ser tão autolimitada quanto previamente se acreditava. Embora no passado séries de casos sugerissem que a recuperação da função renal ocorria independentemente do uso de corticoides sistêmicos, dados mais recentes, incluindo estudos com biópsias seriadas, indicam que pode ocorrer recuperação renal incompleta e progressão para doença renal crônica^15,16^. Este caso reforça a importância de uma intervenção farmacológica precoce em adultos com LRA significativa.

A uveíte tende a responder à corticoterapia; contudo, as recorrências são frequentes e podem ocorrer de forma independente da atividade da doença em nível renal. Neste caso, após a suspensão da corticoterapia sistêmica, observou-se o aparecimento de inflamação ocular no olho contralateral, de forma assintomática. A tomografia de coerência óptica e a angiografia fluoresceínica não revelaram alterações relevantes. Foi iniciada prednisolona tópica, com boa resposta clínica.

Os corticoides tópicos e os agentes cicloplégicos constituem a primeira linha de tratamento da uveíte anterior; formas graves da doença ou a presença de uveíte não anterior justificam a utilização de corticoterapia sistêmica ou terapia imunomoduladora poupadora de corticoides. Esses agentes, que incluem metotrexato, azatioprina ou micofenolato de mofetil, são também utilizados como agentes de segunda linha quando o envolvimento renal não responde adequadamente à corticoterapia isolada. Os inibidores do TNF-alfa demonstraram eficácia em casos graves ou refratários ao tratamento^17^.

### Prognóstico e Seguimento

Neste caso, a creatinina sérica melhorou de 3,54 mg/dL na apresentação para 1,61 mg/dL após a conclusão da corticoterapia, e, posteriormente, para 1,15 mg/dL aos 10 meses de seguimento (Figura 3), com normalização da relação albuminúria/creatininúria (30 mg/g) e resolução da glicosúria. Essa evolução favorável é consistente com o grau leve de fibrose intersticial observada na biópsia.

**Figura 3:** Evolução dos níveis de creatinina sérica (linha contínua) e do tratamento com corticosteroides durante o seguimento.

O prognóstico na síndrome TINU é globalmente favorável quando o tratamento é iniciado precocemente. Diversos fatores influenciam o prognóstico, sendo a idade adulta no momento do diagnóstico, a presença de uveíte posterior ou panuveíte e a maior cronicidade na biópsia renal fatores associados a um maior risco de DRC^2^. As crianças apresentam mais frequentemente recorrência de uveíte, mas menor risco de lesão renal persistente^2^. A disfunção renal permanente ocorre em até 15% dos pacientes, enquanto complicações oculares crônicas (mais frequentemente sinéquias posteriores) afetam aproximadamente 20%^8,18^. Dessa forma, o seguimento multidisciplinar a longo prazo pela Nefrologia e Oftalmologia é essencial.

## Conclusão

Em conclusão, este caso destaca a importância de manter um elevado grau de suspeita para síndrome TINU sempre que coexistam alterações da função renal e inflamação ocular, quer essas manifestações ocorram de forma concomitante, quer sequencialmente. A associação oculorrenal constitui a principal pista diagnóstica, devendo ser considerada a realização de avaliação oftalmológica em pacientes com LRA de etiologia não esclarecida e sinais de disfunção tubular proximal, independentemente da idade.

A colaboração multidisciplinar entre Nefrologia e Oftalmologia é fundamental para assegurar uma abordagem terapêutica otimizada para os pacientes com síndrome TINU, sendo o diagnóstico precoce um passo essencial para melhorar os desfechos renais e visuais.

## Data Availability

Não foram gerados nem analisados conjuntos de dados durante a preparação deste relato de caso. Todos os dados relevantes encontram-se incluídos no manuscrito.
